# Facial expressivity in dominant macaques is linked to group cohesion

**DOI:** 10.1098/rspb.2024.0984

**Published:** 2024-07-17

**Authors:** J. Whitehouse, P. R. Clark, R. L. Robinson, K. Rees, O. O’Callaghan, C. M. Kimock, C. L. Witham, B. M. Waller

**Affiliations:** ^1^ Department of Psychology, Nottingham Trent University, Nottingham NG1 4FQ, UK; ^2^ School of Psychology, University of Lincoln, Lincoln LN6 7TS, UK; ^3^ Centre for Macaques, Medical Research Council, Salisbury SP4 0JQ, UK

**Keywords:** facial expressivity, facial behaviour, social networks, individual differences, macaques

## Abstract

Social living affords primates (including humans) many benefits. Communication has been proposed to be the key mechanism used to bond social connections, which could explain why primates have evolved such expressive faces. We assessed whether the facial expressivity of the dominant male (quantified from the coding of anatomically based facial movement) was related to social network properties (based on social proximity and grooming) in nine groups of captive rhesus macaques (*Macaca mulatta*) housed in uniform physical and social environments. More facially expressive dominant male macaques were more socially connected and had more cohesive social groups. These findings show that inter-individual differences in facial expressivity are related to differential social outcomes at both an individual and group level. More expressive individuals occupy more beneficial social positions, which could help explain the selection for complex facial communication in primates.

## 1. Introduction

The ability to form and maintain complex and stable societies is pronounced across primates and has played an essential role in the trajectory of human evolution. Societies are formed through repeated interactions between individuals and develop into stable and meaningful relationships over time, ultimately affording social primates with many fitness benefits. These benefits include long-term adaptive advantages such as longevity and offspring survival [1,2] and immediate advantages such as protection from predators, mating opportunities and the ability to locate resources more efficiently [3]. We therefore see strong selection for strategies that maintain cohesive societies and strengthen beneficial social relationships with others. One behaviour proposed to facilitate social bonding directly is facial communication [4], but empirical demonstration of this link has been built purely through comparisons at a species level [5–7]. However, whether any individual variation in facial behaviour (within a species) also has an impact on individual sociality is unknown. Yet, meaningful and functional inter-individual variation is an essential platform for evolutionary change [8] and documenting this variation in related species is fundamental to understanding the evolutionary trajectory of complex facial expression unique to the primates and especially so in humans.

Primate signallers provide a variety of information to receivers through the face [9], including identity [10], kin relations [11], dominance asymmetry [12], benign intent [13], affiliation [14], motivation to play [15,16]; all of which also serves the broad function of reducing uncertainty during social interaction [17]. The ability to reliably predict the future actions of others, and thus allow for appropriate behavioural responses, is an essential aspect of navigating complex social environments [18] and the need for this ability is likely a key component in the evolution of all facial signals [19]. Even facial behaviour not directly associated with affiliation such as threat displays is an important component of maintaining peaceful cohesive societies, as they reduce the need for physical (and often extremely disruptive) aggression and conflict [17].

In humans, we have emerging evidence which suggests that stable inter-individual differences in facial expressivity can directly impact the outcome of their social interactions. More specifically, more facially expressive people (those with more facial movement and higher diversity of movement) tend to be rated as more likeable both by third parties [20] and by their direct social partners [21] and can better negotiate resources in their favour [21]. The reasons why we perceive facially expressive people as more likeable have yet to be fully explored, but one suggestion is that the increased readability of a facially expressive partner signals honesty, which is considered a valuable trait in a social partner [19]. By being more likeable, individuals should be better equipped to build more (and higher quality) friendships with others. Data demonstrating how an individual’s facial behaviour is linked to real-world outcomes such as social network formation is currently lacking, both in humans and non-humans.

In many social primate groups, the behaviour of the highest-ranking individual in the dominance hierarchy often has the greatest influence on the organization of the group [22,23]. The influence of the dominant males on the overall structure of the network has been highlighted experimentally in captive groups of pig-tailed macaques (*Macaca nemestrina*), where their removal significantly increased aggression rate, group instability and disrupted social cohesion [24]. This is in part owing to their key role in policing and regulating third-party social interaction—in which effective communication plays a crucial role [25]. In macaques, social network position is highly predicted by sex [26] and matriline membership [26], where females form matrilineal subgroups which are stable over time (and thus, typically have higher network centrality) and males disperse to unrelated groups (and thus, typically have decreased network centrality and are more peripheral). Therefore, we could expect that the effects of any strategies to integrate and bond with others are more exaggerated in males than females, who are more able to rely on matrilineal bonds for social benefits and less on social strategies.

Our limited understanding of individual variation in facial behaviour is probably owing to methodological constraints [27]. Facial behaviour in primates (and all other animals) is typically studied by observing and quantifying holistic facial configurations (e.g. silent-bared teeth, open mouth threat and play face [15,28]). We now know that the variation of behaviour within these facial expression categories is so great that we could conceptually divide them into multiple independent expressions, each with subtly different social functions [29]. It is therefore plausible that any inter-individual variation in facial expression may be absent at these broad categorical levels but present in the more subtle details of facial movement. Using the facial action coding system (FACS) [30], singular facial movements underpinned by a specific muscle contraction can be quantified through the processing of videos frame-by-frame. MaqFACS (FACS modified for use in macaques [31]), allows for an objective measure of 17 unique facial muscle movements (or ‘action units’) and enables the measurement of facial expressivity in fine-grained detail previously unobtainable.

Here, we measured the social network properties of nine social groups of rhesus macaques (*Macaca mulatta*) and assessed how individual and group-level social network attributes are related to the varying facial behaviour of the groups’ alpha males. We predicted that expressive males would have higher quality social connections to others as better communication should increase their ability to better navigate their social environment, and thus occupy a more central position in their group’s affiliation network. We also predicted that the expressivity of the alpha male would be linked to group cohesion, with more expressive males (those producing higher quantity or higher diversity of facial action units) inhabiting more socially bonded and stable groups. Finally, in an exploratory analysis, we assessed if there were any particular facial movements, or combinations of facial movements, that were especially linked with the individual’s social network position.

## 2. Material and methods

### (a) Subjects and housing

Subjects consisted of nine males from different social groups of rhesus macaques (*Macaca mulatta*). The nine groups are composed of a total of 66 adults (including the nine subject males), with a mean of 7.33 (range: 6–9) adults per group. Although juveniles and infants were present in all groups, they were not considered in the calculation of social attributes (as their social networks are highly dependent on the stage of weaning). All animals were housed at the Medical Research Council’s Centre for Macaques (CFM), Salisbury, UK, in nearly identical settings. Each animal could freely access two adjacent rooms; an indoor space (3.5 × 8 × 3 m) equipped with a large outdoor-facing window and enriched with climbing structures, feeding puzzle boxes and other enrichment devices and a second indoor caged area (1.5 × 6 × 3 m). Subjects received a main feed of commercial monkey pellets, fruits and vegetables, including a scatter feed of dried forage mix. All subjects had free access to water. All groups consisted of a single adult male (*n* = 9, who was the focus for our measures of expressivity) with the rest of the group comprising adult females (>3 years old) and the infants and juvenile offspring of these females. A detailed breakdown of group compositions at the time of the study can be found in the electronic supplementary material.

### (b) Data collection

#### (i) Behaviour and proximity sampling

Data were collected between May 2021 and December 2022 by two observers (J.W. and P.R.C.). All animals were followed using focal animal sampling for 10 min observation periods, with scan sampling in 2-min intervals within the focal follow [32]. Animal Observer software for the iPad was used to collect all behavioural data [33]. All observations were made from a viewing window looking into the enclosure. No animals were followed twice within the same day and follows were terminated early if animals were out of sight for longer than 2 min (but any data collected to that point were retained). We recorded all occurrences of affiliative behaviour (social grooming, embracing and approaches), self-directed behaviour (self-grooming, scratching and yawning) and other non-social activities (foraging). During each 2-min scan interval, absolute proximity was estimated (in metres) between the focal and all other group members. In order to minimize the potential for subjective bias when estimating interindividual proximity, data were collected using the visual map interference on Animal Observer software for iPad. During a proximity scan, observers were required to move nodes (representing each individual around a virtual map of the enclosure. This allows for an objective and non-cognitively demanding measure of proximity, which is extracted from the distance between nodes at a later date. All occurrences of agonistic behaviour in the group (contact and non-contact conflict, displacement) were recorded using *adlibitum* sampling. On average, the 66, comprising nine social groups were observed for on average 3.4 h (±1.4) of focal follows and received on average 103 (±44) scans. More detailed definitions of behaviour can be found in the electronic supplementary material.

#### (ii) Facial behaviour

In addition to our behavioural data, close-up video footage was collected of the male of each group from the enclosure viewing window. This footage was recorded to quantify facial expressivity in detail. As individuals were often obscured by objects in the enclosure, facing away from the observer, or were not visible owing to poor lighting conditions (e.g. reflections off the glass or in a darker area of the enclosure), videos were collected opportunistically when conditions which optimized the quality of video footage were met. Video clips were recorded for a maximum of 10 min per observation period; however, observations were terminated before this if an individual’s face went out of sight. Video footage was collected in this way until approximately 2 h of raw video footage was collected for each subject. Videos were collected with a Panasonic HDC-SD700, filmed at a frame rate of 50FPS and saved as .mp4 video files.

### (c) Social measures

#### (i) Dyadic sociality Index

From our scan data, we generated dyadic proximity matrices for each group. Proximity scores for dyad (AB) were calculated as the total summed distance observed between individuals A and B, divided by the total number of scans conducted on A + B. This measure results in an average distance that each individual was observed from every other individual (with a lower score meaning closer average proximity from each other). If during a scan, individuals of a dyad were in separate spaces (e.g. one indoors and one outdoors), their proximity was set as maximum inter-individual distance (which is the greatest distance individuals could be seen apart owing to enclosure constraints; 6 m). From our focal data, we generated dyadic grooming matrices for each group. Grooming scores for dyad AB were calculated as the total summed time individuals AB were observed in a grooming interaction, divided by the total focal time of A + B. This measure results in an average amount of grooming observed between each potential dyad.

Finally, we calculated a dyadic sociality index (DSI) [34], for each dyad from a composite of these two (proximity and grooming) measures. Before DSI was calculated, proximity scores were reversed by subtracting all values from the maximum inter-individual distance. The purpose of this was to make higher values reflect stronger associations (as comparable with the grooming measure). For each sociality measure (*d*), the score within a dyad AB (*f_ab_*) was divided by the mean across all dyads ( $\overset{-}{f_{i}}$ ). The purpose of this is to attribute the same weighting to each sociality measure. The resulting values were summed across both measures and then divided by 2 (the total number of measures). The resulting value for each dyad is the DSI for dyad AB ( ${DSI}_{ab}$ )


$$Dyadic\;\;Sociality\;\;Index\;\;for\;\;Dyad\;\;AB=\;{DSI}_{ab}=\;\frac{\sum_{i=1}^{d}\frac{fiab}{\overset{-}{fi}}}{d}$$

#### (ii) Social network analysis

**Figure 1.**
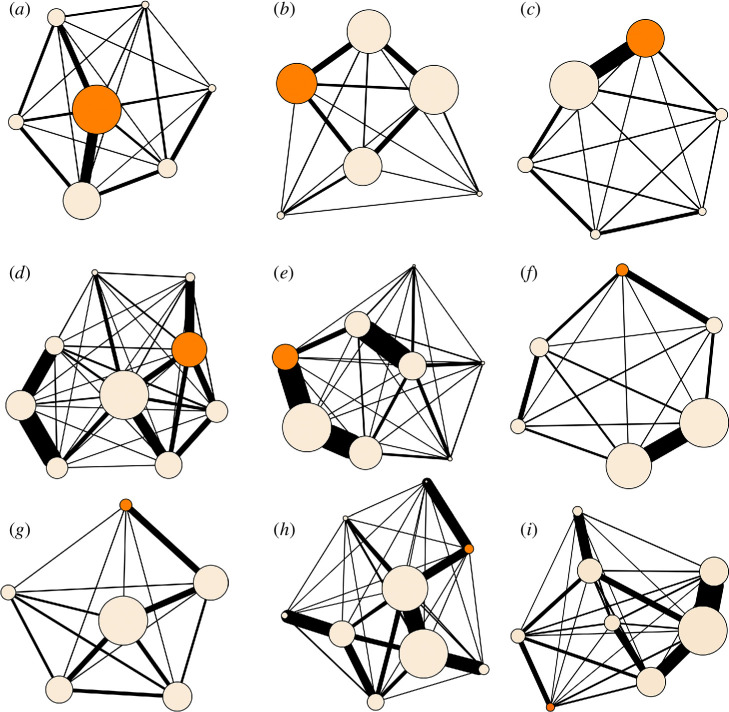
Sociograms of social groups. (*a*) Group BTJ, (*b*) group VNT, (*c*) group YOK, (*d*) group ZODI, (*e*) group STAR, (*f*) group ASAM, (*g*) group BAG, (*h*) group PLUM and (*i*) group WILL. Nodes represent individuals, Edge thickness represents social connections as calculated through DSI. Orange nodes represent the single adult male of each group, off-white nodes represent the adult females. All sociograms were built using *igraph* for *R* [35].

For each group, we calculated individual (for the male subject) and group-level social network attributes from DSI matrices. A visualization of the nine social groups can be found in figure 1.

For our individual social network measures, we calculated the eigenvector centrality and the betweenness centrality of each male using functions *eigen_centrality* and *betweenness* from the R package *igraph* [35]. Eigenvector centrality can be interpreted as the social capital of an individual or the potential availability of social resources. Individuals with higher eigenvector centrality are more well connected to others and have connections with those who are also highly connected. Betweenness refers to the flow of information through a network and specifically, is the number of times that individual is travelled through when calculating paths linking all dyads [36,37]. Those with high betweenness play an important role in linking social gaps between other individuals. We measured nodal strength (the sum of all weighted connections to each male) and network strength (the sum of all weighted connections within the group). As we needed to compare between groups, we used non-standardized affiliation matrices (i.e. those featuring raw proximity and grooming scores) to calculate nodal strength (the sum of all ties/connections for each male).

For our group-level social network measures, we calculated network strength (the sum of all ties across the group) from proximity and grooming scores. We also calculated group modularity with function *Modularity* for *igraph* using a *cluster_walktrap* community detection. Modularity measures the degree to which a network could be divided into subgroups (or clusters [38]). A modularity score of 0 signifies random and even assortment, a modularity score towards 1 signifies the presence of a community structure. Finally, we calculated a degree centralization score for each group. Centralization is a method of calculating a network-level measure by quantifying the distribution of centrality scores throughout a group [39]. A higher centralization score signifies an uneven distribution of centrality scores throughout a group, with a smaller number of influential individuals. A lower centralization score signifies an even distribution of centrality scores throughout a group, where the centrality of each member is more similar. To normalize this score across groups, a theoretical maximum centrality score was calculated per group using the *centr_eigen_tmax* function for *igraph,* this score is then fed into the *centralized* function for *igraph* to generate the centralization score. Incorporating this theoretical maximum score into our calculations allows for comparisons across groups of different numbers of individuals.

#### (iii) Dominance

From data collected during ad libitum observations of agonistic interactions, we calculated individual and group-level hierarchy attributes using STEER (Steepness estimation with Elo-rating; R package *EloSteepness* [40]). Using STEER, we extracted the *summed winning probability* of each male, which is the sum of the expected winning probabilities between the male and each potential opponent in the group, or in other words, a measure of the likelihood of an individual winning a single agonistic interaction with a random partner (for more details see [40]). This score was normalized by dividing the final *summed winning probability* by the size of the individual’s group. In addition, we also extracted the hierarchy steepness for each group. Steepness quantifies the degree of competition within a social group, with steeper hierarchies consisting of more strict, asymmetrical dominance relationships. In addition to the steepness, we also calculated the overall aggression rate of the group (the number of aggressive interactions observed in the group, divided by observation time). This measure was also normalized by dividing the value by the size of the group.

### (d) Expressivity measures

The video footage collected of our male subjects (*n* = 9) was coded using MaqFACS (facial action coding system modified for use with macaques [31]), by six certified MaqFACS coders. In the MaqFACS system, individual facial movements (or action units/AUs) are coded, each of which based on an underlying muscle movement. This allows for an objective and detailed quantification of facial movement. For each video, 17 AUs were coded and the onset (when the movement was first seen as active) and offset (when any muscle activation could no longer be reliably seen) of each movement were coded whenever it was present. AU43/eyes closed was excluded as this movement is usually an indication of sleep rather than having any communicative function. All video coding was conducted in the observation software BORIS (Behavioural Observation Research Interactive Software [41]). A full breakdown of AUs coded and their descriptions can be found in table 3. Whenever individuals were chewing or eating, ‘feeding’ was coded (additional action descriptor AD50a) and any AUs associated with mastication or the manipulation of food (e.g. AU25/lips parted) were ignored. During these periods, any non-feeding-related AUs were coded as normal. When faces were obscured or where coding would be unreliable, AUX/out of sight was coded and FACS coding was paused. A true observation time was coded from these data (total video footage – total AUX duration). After accounting for this, we were left with on average 87.3 (±23.2) min of quantified facial movement per individual. In total, 10 158 facial movements were quantified (a mean of 1128.7 ± 360.6 per individual).

The current context observed in the video was also recorded. For example, we coded if the animal was currently engaged in affiliative behaviour (with conspecifics), aggressive behaviour (with conspecifics), affiliative behaviour (towards the observer), aggressive behaviour (towards the observer), low vigilance (scanning eye movements only), high vigilance (including head and body movements). A full breakdown of all contexts and their definitions can be found in the supplementary material (electronic supplementary material 3). The purpose of this additional contextual information was to appropriately nest our AU data into the context in which it occurred in order to reduce any bias in our expressivity measures owing to an unequal observation of contexts seen across individuals (see calculation of these measures in table 1).

**Table 1.** Calculations and descriptions of the four facial expressivity measures. Each measure was calculated per individual, per context. The final expressivity measures for each individual represents the average (mean) score across contexts.

calculations	description
$overall\;AU\;rate=\;\sum_{i=1}^{C}\frac{f_{i}\;}{t_{i}\;C}$	The numbers of facial movements observed per unit time (s). *f i*s the number of AUs observed*, t* is the observational time and *C i*s the number of contexts observed.
$overall\;AU\;duration=\;\sum_{i=1}^{C}\frac{d_{i}\;}{t_{i}\;C}$	The percentage of observation time where an AU occurred. *d* is the sum of AU durations, *t* is the observational time, *C* is the number of contexts observed.
$H_{c}=-\sum_{i=1}^{S}P_{i}log\left(P_{i}\right)$ $D_{c}=e^{H_{c}}$ $AU\;diversity=\;\sum_{i=1}^{C}\frac{D_{c}}{C}$	$H_{c}$ is Shannon’s information for data within each observed context. AU diversity (*D*) represents the evenness of AU representation. This measure is maximized when all AUs are evenly present throughout an individual’s facial behaviour. *S i*s the number of AUs for a given individual, $P_{i}$ is the ratio between the frequency of each facial expression and total AU frequency, and *C* is the number of contexts observed.
$individual\;AU\;proportion=\;\frac{AU_{x}}{f}$	The ratio between the frequency of AU_x_ and total frequency of all AUs.

Four expressivity measures were calculated: AU rate, AU duration (controlled for observation time), AU diversity [42] and AU proportion—a breakdown of how these scores were calculated and their definitions can be seen in table 1. We calculated each expressivity measure per individual per context and averaged the score across contexts. This was to account for the fact observation time of each context varied across individuals and we did not want context-specific facial movement to impact our final measures (e.g. to ensure those individuals engaged in more social interaction were not erroneously labelled as more expressive). Through this method, each context contributed equal weighting towards the final expressivity score for each individual. To confirm that the generation of our scores was not significantly impacted by the inclusion of contexts with very low observation time, we regenerated our expressivity measures excluding contexts with less than 60 s of data. These regenerated scores were significantly correlated with our original scores, suggesting that our expressivity measures are robust against any potential issue this may cause. A full description of this analysis and results can be found in the supplementary materials (electronic supplementary material 4). Intercoder reliability was conducted using an intraclass correlation using 10 randomly selected videos (function ICC in package *psych* [43]) which were coded independently. This revealed high average agreement across the FACS coding of our coders (average ICC: 0.75, CI: 0.63–0.84, *p <* 0.001). All coders were also FACS certified before coding; certification is obtained by achieving high reliability with a master FACS coding task on rhesus macaques (Wexler’s agreement for all coders >0.70).

#### (i) Statistical analysis

We built generalized linear models (GLM with Gaussian error structure) using function *glm* for R studio [44]. As we only have nine data points per measure (one per male), all models were built with a single predictor of interest to reduce the risk of overfitting to the dataset.

We generated models assessing the relationship between three expressivity measures and various social measures. Each expressivity measure acted as predictors in our models: (i) AU rate, (ii) AU duration, and (iii) AU diversity. We tested the relationship of these predictors with 11 outcome variables, all of which relate to individual or group-level social attributes. Individual attributes included (i) eigenvector centrality, (ii) betweenness centrality, (iii) nodal strength from grooming, (iv) nodal strength from proximity and (v) winning probability. Group-level attributes included (vi) centralization, , (vii) network strength from grooming, (viii) network strength from proximity, (ix) modularity, (x) group aggression and (xi) hierarchical steepness. Each model was compared with a null model containing only the outcome variable and the intercept, to see if the inclusion of each predictor significantly improved model fit. These comparisons were made using a likelihood ratio test (LRT) using function *lrtest* from R package *lmtest* [45].

In a further exploratory analysis, we built models to explore the impact of each of the 17 individual action units on eigenvector centrality. We chose this social network measure for further exploration, as the direct impact of this measure on fitness is well understood [1]. These exploratory analyses will help us unpack whether it is broad expressivity across all facial behaviours that is more predictive of social networks, or if there are any specific movements or combinations that are of importance. In each of these models, the predictor was the AU proportion of the AU of interest (i.e. what proportion of overall facial movement does this AU represent) and the outcome variable was the eigenvector centrality of the individual. Similarly, each model was compared with a null model containing only the outcome variable and the intercept (using an LRT) to see if the inclusion of each predictor significantly improved model fit. For visualization purposes, we also calculated the *R* ^2^ of each model (or the proportion of the variation in eigenvector centrality that is predicted to be associated with each AU). As AUs are likely to be interrelated, further exploratory analysis incorporating the co-variation of these individual AU movements was conducted to avoid the misinterpretation of multiple effects as independent. To do this, we reduced our AU proportion data down into related clusters of AUs using k-means clustering. First, we determined the optimal number of cluster centres (*k*) by generate a silhouette score for *k* values 2–17 (i.e. number of AUs, package *Cluster* [46]). Silhouette score compares within-cluster similarity of a data point to other similar clusters, with the *k*-means with the highest score representing the optimal value of *k*. AU data were aggregated depending on subsequent cluster membership and were then subjected to the same analysis above conducted on individual AUs (see electronic supplementary material for more detail about the cluster analyses.

As our analytical approach was largely exploratory, we chose not to adjust our significance threshold to control for multiple tests as these adjustments are difficult to employ in exploratory work without significant risk of type 2 errors [47]. We therefore approach significant results with additional caution.

## 3. Results

### (a) Relationship between expressivity measures and social attributes

**Figure 2.**
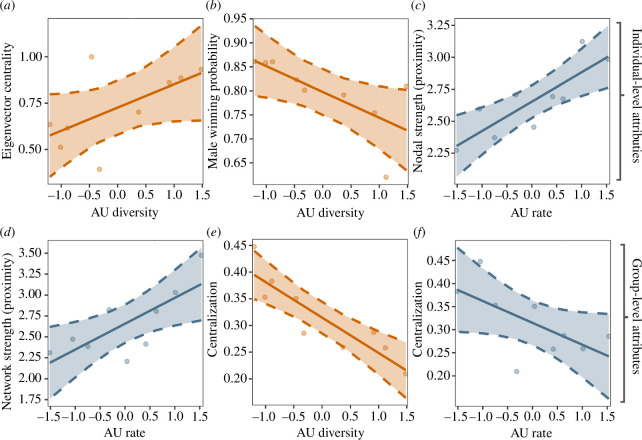
Notable (significant) relationships. (*a–c*) The relationship between expressivity measures and individual-level social network attributes. (*d–f*) The relationship between expressivity measures on group-level attributes. Plots with data coloured in orange represent relationships with AU diversity, and plots with data colours in blue represent relationships with AU rate. Plots c and d are based on a reversed proximity score to depict stronger relationships as a higher value. All expressivity measures were *z*-scored so they can be directly compared visually. Error bars represent standard errors.

We found the males’ eigenvector centrality was predicted by their AU diversity (or in other words, how evenly distributed an individual’s AU usage is; LRT, null versus full model: *χ* ^2^ = 4.18, *p* = 0.041; figure 2*a*), the higher an individual’s diversity of facial movement, the more centrally positioned they were in their social group. This was not predicted by our other measures; AU rate (number of facial movements per unit time) or AU duration (amount of time any facial movement was present on the face, controlled for observation effort, LRT: *χ* ^2^ = 0.001, *p* = 0.976 and *χ* ^2^ = 0.224, *p* = 0.636, respectively). AU diversity also predicted the male’s winning probability (i.e. a measure of their competitive success as calculated from their Elo-rating) (LRT: *χ* ^2^ = 6.299, *p* = 0.012; figure 2*b*), which was negatively associated, males with more diverse facial movements were less likely to win aggressive interactions. The males’ nodal strength (calculated using proximity) was predicted by AU rate (LRT: *χ* ^2^ = 10.78, *p* = 0.001); expressive males had stronger proximity connections with others. Network centralization was predicted by both AU rate and AU diversity (LRT: *χ* ^2^ = 4.71, *p* = 0.030 and *χ* ^2^ = 14.56, *p* ≤ 0.001, respectively; figure 2*c* and *f*), males with higher scores in these expressivity indices had less centralised social groups (i.e. a more uniform distribution of social connections). AU duration, however, did not predict group centralization (LRT: *χ* ^2^ = 3.63, *p* = 0.057). The males’ network strength was predicted by AU rate (LRT: *χ* ^2^ = 7.56, *p* = 0.006, respectively; figure 2*d* and *e*); the groups of expressive males had stronger proximity connections overall. No expressivity measures predicted betweenness, group modularity, network strength calculated from grooming interactions, overall group aggression or the steepness of the hierarchy. A full list of results can be found in table 2.

**Table 2.** Results of likelihood ratio tests. Models containing the predictor of interest were compared with a null model containing intercepts only. Significant *p*-values (*p* < 0.05) are highlighted in bold.

	predictor					
	AU rate		AU duration		AU diversity	
outcome variable	*χ* ^2^	*p*	*χ* ^2^	*p*	*χ* ^2^	*p*
*individual level attributes*						
eigenvector centrality	0.00	0.976	0.224	0.636	4.182	**0.041**
betweenness	1.094	0.296	1.305	0.253	0.612	0.434
strength (nodal and grooming)	0.630	0.427	2.598	0.107	2.042	0.153
strength (nodal and proximity)	10.78	**0.001**	2.480	0.115	2.071	0.150
winning probability	2.768	0.096	0.009	0.925	6.299	**0.012**
*group-level attributes*						
centralization	4.714	**0.030**	3.628	0.057	14.56	<**0.001**
strength (network and grooming)	1.730	0.189	3.471	0.062	1.534	0.215
strength (network and proximity)	7.556	**0.006**	3.591	0.058	0.877	0.349
modularity	0.226	0.634	0.006	0.939	0.669	0.407
group aggression	0.408	0.523	1.561	0.212	0.022	0.883
hierarchy steepness	0.137	0.711	0.577	0.447	0.734	0.392

### (b) Relationship between individual action units and eigenvector centrality

**Figure 3.**
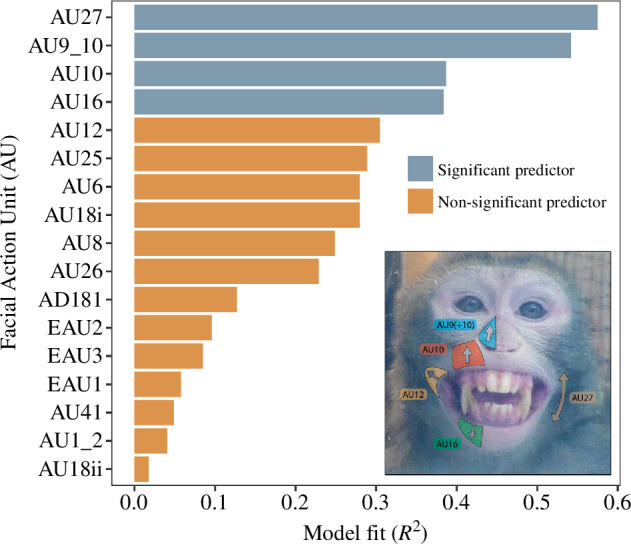
Comparison of model fit: individual AUs. Each model contained AU proportion as the predictor variable, and eigenvector centrality as the outcome variable. Models ranked by their predictive value (*R* ^2^). Bottom right panel: example of AUs acting on the face of a macaque; coloured outlines show area on the face affected by the movement.

To further explore the impact of facial behaviour on eigenvector centrality, we assessed whether any specific facial movements were driving this effect. As a first step, we looked at AUs independently, without assuming any prior assumption of co-occurrence between AUs. Four lower face movements out of the 17 potential AUs predicted increased eigenvector centrality; mouth stretch (AU27: LRT: *χ^2^* = 7.698, *p* = 0.006), nose wrinkle and upper lip raiser (AU9+10: *χ^2^* = 7.026, *p* = 0.008), upper lip raiser in isolation (AU10: *χ^2^* = 4.399, *p* = 0.036) and lower lip depressor (AU16, *χ^2^* = 4.364, *p* = 0.037). All other action units did not significantly predict eigenvector centrality, however overall, lower face movements appeared to be in general, more predictive than upper face/ear movements when ranked in order of predictive value (figure 3). We acknowledge that these findings may not be independent from each other as facial movements as often expressed as combinations of AUs, so their interpretation as individual effects should be approached with caution. This is addressed in the subsequent analyses. A full list of results can be seen in table 3.

**Table 3.** Predictive value of each individual AU, results of likelihood ratio tests. Models containing the predictor of interest were compared with a null model containing intercepts only.

outcome variable: eigenvector centrality					
model^a^	descriptive	location^b^	ΔAICc	X^2^	*p*
AU27	mouth stretch	lower	0.00	7.698	**0.006**
AU9+10	nose wrinkle+upper lip raiser	lower	0.67	7.026	**0.008**
AU10	upper lip raiser	lower	3.30	4.399	**0.036**
AU16	lower lip depressor	lower	3.33	4.364	**0.037**
AU12	lip corner puller	lower	4.42	3.274	0.070
AU25	lips parted	lower	4.63	3.068	0.080
AU18i	true pucker	lower	4.74	2.962	0.085
AU6	cheek raiser	upper	4.74	2.960	0.085
AU8	lips towards each other	lower	5.12	2.580	0.108
AU26	jaw drop	lower	5.36	2.336	0.126
AD181	lipsmacking	lower	6.38	1.222	0.269
EAU2	ear elevator	ears	6.79	0.903	0.342
EAU3	ear flattener	ears	6.90	0.802	0.370
EAU1	ears forward	ears	7.16	0.541	0.462
AU41	glabella lowerer	upper	7.24	0.453	0.501
AU1+2	brow raiser	upper	7.33	0.372	0.542
AU18ii	outer pucker	lower	7.53	0.163	0.686

^a^ Each model contained AU proportion as the predictor variable.

^b^ AU location on the face (upper face, lower face or ear movement). ΔAICc refers to the change in AICc between the best-fitting model (AU27) and the rest of the models.

### (c) Relationship between AU clusters eigenvector centrality

As action units have a high likelihood of co-variation and are often expressed in combinations of behaviour, we reduced our individual AU proportion data to clusters of action units with high association using a *K*-means clustering analysis. We then assessed the link between these clusters and centrality. An initial silhouette analysis determined that the optimal *K* value (or optimal number of cluster centres) was 7. AU membership for each of the seven clusters was as follows: (i) AU25 + AU26, (ii) EAU2 + AU18 i, (iii) AU17 + EAU3, (iv) AU41 + AU1+2, (v) AU9&10 + AU10 + AU16 + AU27 + AU12 + AU8 + AU6, (vi) AD181 and (vii) EAU1 + AU18 ii.

A single AU cluster (cluster 5) significantly predicted centrality (LRT: *χ* ^2^ = 5.68, *p* = 0.017). This cluster contained all four action units which emerged as significantly predicting centrality in the previous analysis (AU27, AU9 + 10, AU9 and AU16), suggesting these are not independent effects but are linked. However, three additional AUs attributed to this cluster were not significantly linked to centrality when assessed on their own (AU12, AU8 and AU6), suggesting that not all components of this cluster are important in regard to their centrality benefits. A full list of results can be seen in table 4.

**Table 4.** Predictive value of each AU cluster, results of likelihood ratio tests. Models containing the predictor of interest were compared with a null model containing intercepts only. AUs marked with an asterisk were individually significant in prior analysis.

outcome variable: eigenvector centrality				
AU cluster^a^	AUs	ΔAICc	*χ* ^2^	*p*
cluster 5	AU9+10*, AU10*, AU16*, AU27*, AU12, AU8, AU6	0	5.682	**0.017**
cluster 2	EAU2, AU18i	2.818	2.864	0.091
cluster 1	AU25, AU26	2.853	2.829	0.093
cluster 6	AD181	4.46	1.222	0.269
cluster 3	AU17, EAU3	4.964	0.717	0.397
cluster 4	AU41, AU1+2	5.221	0.46	0.497
cluster 7	EAU1, AU18ii	5.246	0.435	0.509

^a^ Each model contained the proportion of AUs observed in that cluster as the predictor variable. ΔAICc refers to the change in AICc between the best-fitting model (cluster 5) and the rest of the models.

## 4. Discussion

This study links social properties with individual differences in facial expressivity in a non-human primate and provides evidence to suggest facial behaviour plays a key role in modulating both egocentric and group-level social connectivity. Being more expressive seemed to offer social benefits to the males of the nine single-male groups, as they were more well connected to others. Greater facial expressivity in these individuals appears to also have a broader social influence on the cohesion of their group, which was more tightly organized in space. Both a greater diversity of facial behaviour and a greater overall quantity of facial movements were seen in males living within more decentralized societies, hinting at the possibility that facial behaviour is linked to individual leadership and/or social style in primates. The more socially tolerant males in our sample, i.e. those with less asymmetry in their conflict outcomes, were also characterized by higher expressivity indices, further supporting the long-standing hypothesis that more tolerant social strategies require more complex communicative tools to manage. The hypothesis linking social tolerance with communicative complexity is also relevant for explaining the relationship between the diversity of facial movement and winning probability in conflicts in the studied males (with more expressive males also showing less conflict asymmetry [6]).

Males with higher diversity of facial movement were better connected to their social group; these males’ social groups also showed comparatively less network centralization (i.e. social connectivity was more evenly distributed throughout their members). Macaque males often occupy a more peripheral position in the social network, with male–female relationships being weaker compared with female–female relationships [26]. In groups with more expressive males, males were comparatively less peripheral and overall, social connections were more uniform. Although a higher diversity of facial movements is not necessarily indicative of better communication, these data suggest that this facet of expressivity provides an advantage when forming and maintaining social connections to others. Crested macaques (*Macaca nigra*), who are characterized by their social tolerance and relatively higher social bonding [48], also display enormous diversity in their affiliative facial signalling and incorporate AU configurations that vary depending on the specific affiliative context [29,49]. The positive impact of facial movement diversity on social connectivity in the current study may be occurring when individuals are using communicative strategies more similar to these more socially tolerant species and consequently have similar advantages during social bonding.

The idea of social tolerance is also relevant when we look at the relationship between facial movement diversity and conflict-winning probability. Males with more diverse facial behaviour were also those with less asymmetric conflict interactions (i.e. conceded more competitive interactions) providing further support that those individuals may have a more tolerant and less despotic social style [48]. Or instead, it could be that having a higher diversity of facial signals and less stereotyped and fixed signals incurs an unavoidable competitive cost to the signaller. Clear and unambiguous signalling in agonistic and dominance interactions is known to be advantageous as it reduces the likelihood of miscommunication during risky situations and prevents escalated aggression [7,50]. Or alternatively, it could be that more subordinate individuals display more diverse communication to navigate the potentially more complex social world with more varied dominant relationships. Whatever the mechanism is at play, these differing social outcomes—to gain either affiliative or competitive advantages—appear to be linked to an individual’s facial behaviour. If varying styles of facial expressivity offer individual’s various social benefits, this may help explain why stable individual differences in facial expression production are able to evolve.

Not just overall facial movement diversity, but some specific muscle movements and clusters of movements predicted an increase in centrality when they were used at a higher proportion in an individual’s repertoire. In our exploratory analyses of individual facial movements, four movements were associated with centrality, all of which control teeth exposure; the raising of the upper lip to expose the top teeth (accompanied with or without a nose-wrinkle: AU9 + 10 and AU10), the lowering of the bottom lip to expose the bottom teeth (AU16) and the wide opening of the mouth (AU27). Although other movements were not significantly predictive of centrality, nearly all lower face muscle movements had higher predictive value than upper face or ear movements. These four significant movements co-varied and formed part of the same cluster, also containing AU12, AU6 and AU8. This cluster predicted the centrality of the male, suggesting the previous results on individual AUs are not necessarily independent findings but are all related to the effect of this combination of AUs. The clustering of AU12, AU9 + 10, AU10 and AU16 is quite typical of stereotyped silent-bared teeth (or ‘fear-grin’) expressions in macaques [31], and the incorporation of AU27 during bared teeth displays can be associated with affiliative play contexts [29]. In macaques (and many other primate species) these bared-teeth displays are used typically as signals of appeasement and submission in more despotic species [28,51] and during social bonding and affiliation in tolerant species [29]. It could be that individuals who are more expressive in these bared-teeth displays are navigating these affiliative or submissive interactions more effectively, which could subsequently allow them to occupy more favourable social network positions if it is aiding the strengthening of relationships.

The total amount of facial movement observed in the males (regardless of the composition of the AUs produced) predicted the cohesion of the group on the whole. When the top-ranking male was a more expressive individual (i.e. in this case, produced more movement) the individuals of the group were on average closer to each other in space. This suggests that facial behaviour is not only related to the structure of the network, but groups with expressive males also had comparatively stronger social bonds. The actions and behaviours of top-ranking males is known to directly impact the structure of macaque social networks, owing to the key role these individuals have in moderating third-party interactions [24]. Although these data at present are unable to directly assess the impact of policing and other third-party behaviours, it could be that greater expressivity is linked with a more successful moderation of these interactions. An individual who is able to communicate more effectively may consequently be able to assist better in the reduction of conflict in the group through more successful intervention.

These findings contribute an important step towards an understanding of the adaptive value of facial behaviour beyond their immediate proximate function [52]. For the first time, we have shown meaningful variation in facial behaviour within a species is directly related to individual social outcomes. However, we emphasize some caution around these findings and acknowledge that more investigation is needed. Without the incorporation of any experimental manipulations, we cannot be certain about causality and its direction in our findings. We interpret our data as showing behavioural impacts on sociality (as we believe this is the most parsimonious explanation), but it is possible that we are instead observing a social impact on behaviour. We also acknowledge limitations in our sample size, which do not allow for an overly robust or confident exploration of individual differences. Similarly, our groups are limited to a single male per group, which is unusual for wild macaques which typically live in multi-male, multi-female societies [48]. On the one hand, this makes our results less generalizable to these wild populations, however, on the other hand, allows for more controlled comparisons between groups by accounting for the significant impact male–male competition has on a male’s social environment [53]. Although we found no effect of expressivity on some of our other social network metrics (e.g. betweenness and modularity), it is difficult to conclude if these are true negatives or if the size of the groups is not large enough to identify these effects. Social network metrics such as betweenness and modularity are more prevalent as group size increases, and meaningful subgroup formation may not be detected with these small group sizes. However, despite these limitations, we argue that systematic comparison across nine social groups of primates in uniform physical and social environments is rare and provides an important scientific contribution.

## Data Availability

All data and associated files are available on the Open Science Framework [54]. Supplementary material is available online [55].

## References

[1] Silk JB , Alberts SC , Altmann J . 2003 Social bonds of female baboons enhance infant survival. Science **302** , 1231–1234. (10.1126/science.1088580) 14615543

[2] Silk JB , Beehner JC , Bergman TJ , Crockford C , Engh AL , Moscovice LR , Wittig RM , Seyfarth RM , Cheney DL . 2010 Strong and consistent social bonds enhance the longevity of female baboons. Curr. Biol. **20** , 1359–1361. (10.1016/j.cub.2010.05.067) 20598541

[3] Silk JB . 2007 The adaptive value of sociality in mammalian groups. Phil. Trans. R. Soc. B **362** , 539–559. (10.1098/rstb.2006.1994) 17363359 PMC2346516

[4] Dobson S . 2012 Face to face with the social brain. Phil. Trans. R. Soc. B **367** , 1901–1908. (10.1098/rstb.2011.0224) 22641828 PMC3367705

[5] Dobson SD . 2009 Socioecological correlates of facial mobility in nonhuman anthropoids. Am. J. Phys. Anthropol. **139** , 413–420. (10.1002/ajpa.21007) 19235791

[6] Dobson SD . 2012 Coevolution of facial expression and social tolerance in macaques: facial expression and social tolerance. Am. J. Primatol. **74** , 229–235. 24006541 10.1002/ajp.21991

[7] Rincon AV , Waller BM , Duboscq J , Mielke A , Pérez C , Clark PR , Micheletta J . 2023 Higher social tolerance is associated with more complex facial behavior in macaques. Elife **12** , RP87008. (10.7554/eLife.87008) 37787008 PMC10547472

[8] Badyaev AV . 2011 Origin of the fittest: link between emergent variation and evolutionary change as a critical question in evolutionary biology. Proc. R. Soc. B **278** , 1921–1929. (10.1098/rspb.2011.0548) PMC3107662 21490021

[9] Waller BM , Kavanagh E , Micheletta J , Clark PR , Whitehouse J . 2022 The face is central to primate multicomponent signals. Int. J. Primatol. **45** . (10.1007/s10764-021-00260-0)

[10] Micheletta J , Whitehouse J , Parr LA , Marshman P , Engelhardt A , Waller BM . 2015 Familiar and unfamiliar face recognition in crested macaques (Macaca nigra). Open Sci. **2** , 150109. (10.1098/rsos.150109) PMC4453246 26064665

[11] Mateo JM . 2009 Kinship signals in animals. In Encyclopedia of neuroscience (ed. LR Squire ), pp. 281–289. Oxford, UK: Academic Press.

[12] Tibbetts EA , Pardo-Sanchez J , Weise C . 2022 The establishment and maintenance of dominance hierarchies. Phil. Trans. R. Soc. B **377** , 20200450. (10.1098/rstb.2020.0450) 35000449 PMC8743888

[13] Waller BM , Dunbar RIM . 2005 Differential behavioural effects of silent bared teeth display and relaxed open mouth display in chimpanzees (Pan troglodytes). Ethology **111** , 129–142. (10.1111/j.1439-0310.2004.01045.x)

[14] Petit O , Thierry B . 1992 Affiliative function of the silent bared-teeth display in moor macaques (Macaca maurus): further evidence for the particular status of Sulawesi macaques. Int. J. Primatol. **13** , 97–105. (10.1007/BF02547729)

[15] Preuschoft S . 2000 Primate faces and facial expressions. Soc. Res. **67** , 245–271.

[16] Davila-Ross M , Palagi E . 2022 Laughter, play faces and mimicry in animals: evolution and social functions. Phil. Trans. R. Soc. B **377** , 20210177. (10.1098/rstb.2021.0177) 36126662 PMC9489294

[17] Waller BM , Whitehouse J , Micheletta J . 2016 Macaques can predict social outcomes from facial expressions. Anim. Cogn. **19** , 1031–1036. (10.1007/s10071-016-0992-3) 27155662 PMC4967087

[18] Dunbar RIM . 2020 Structure and function in human and primate social networks: implications for diffusion, network stability and health. Proc. R. Soc. Math. Phys. Eng. Sci. **476** , 20200446. (10.1098/rspa.2020.0446) PMC7482201 32922160

[19] Waller BM , Whitehouse J , Micheletta J . 2017 Rethinking primate facial expression: a predictive framework. Neurosci. Biobehav. Rev. **82** , 13–21. (10.1016/j.neubiorev.2016.09.005) 27637495

[20] Whitehouse J , Milward SJ , Parker MO , Kavanagh E , Waller BM . 2022 Signal value of stress behaviour. Evol. Hum. Behav. **43** , 325–333. (10.1016/j.evolhumbehav.2022.04.001)

[21] Kavanagh E , Whitehouse J , Waller B . 2024 Being facially expressive is socially advantageous. Sci. Rep. **14** . (10.1038/s41598-024-62902-6) PMC11176176 38871925

[22] Drews C . 1993 The concept and definition of dominance in animal behaviour. Behaviour **125** , 283–313. (10.1163/156853993X00290)

[23] Rodriguez-Santiago M , Nührenberg P , Derry J , Deussen O , Francisco FA , Garrison LK , Garza SF , Hofmann HA , Jordan A . 2020 Behavioral traits that define social dominance are the same that reduce social influence in a consensus task. Proc. Natl Acad. Sci. USA **117** , 18566–18573. (10.1073/pnas.2000158117) 32675244 PMC7414064

[24] Flack JC , Girvan M , de Waal FBM , Krakauer DC . 2006 Policing stabilizes construction of social niches in primates. Nature **439** , 426–429. (10.1038/nature04326) 16437106

[25] Flack JC , de Waal FBM , Krakauer DC . 2005 Social structure, robustness, and policing cost in a cognitively sophisticated species. Am. Nat. **165** , E126–E139. (10.1086/429277) 15795848

[26] Sosa S . 2016 The influence of gender, age, matriline and hierarchical rank on individual social position, role and interactional patterns in macaca sylvanus at ‘la forêt des singes’: a multilevel social network approach. Front. Psychol. **7** , 529. (10.3389/fpsyg.2016.00529) 27148137 PMC4834345

[27] Waller BM , Julle-Daniere E , Micheletta J . 2020 Measuring the evolution of facial ‘expression’ using multi-species FACS. Neurosci. Biobehav. Rev. **113** , 1–11. (10.1016/j.neubiorev.2020.02.031) 32105704

[28] Preuschoft S , van Hooff JARAM . 1995 Homologizing primate facial displays: a critical review of methods. Folia Primatol. **65** , 121–137. (10.1159/000156878) 8846993

[29] Clark PR , Waller BM , Burrows AM , Julle-Danière E , Agil M , Engelhardt A , Micheletta J . 2020 Morphological variants of silent Bared‐Teeth displays have different social interaction outcomes in crested macaques (Macaca nigra). Am. J. Phys. Anthropol. **173** , 411–422. (10.1002/ajpa.24129) 32820559

[30] Ekman P , Friesen W . 1978 Facial action coding system. Consultation Psychologists Press.

[31] Parr LA , Waller BM , Burrows AM , Gothard KM , Vick SJ . 2010 Maqfacs: a muscle-based facial movement coding system for the Rhesus Macaque. Am. J. Phys. Anthropol. **143** , 625–630. (10.1002/ajpa.21401) 20872742 PMC2988871

[32] Altmann J . 1974 Observational study of behavior: sampling methods. Behaviour **49** , 227–267. (10.1163/156853974x00534) 4597405

[33] Caillaud D . 2012 Animal observer. A free iPad application for behavioral observations, activity budgets and animal health monitoring. See https://fosseyfund.github.io/AOToolBox/.

[34] Schülke O *et al* . 2022 Quantifying within-group variation in sociality—Covariation among Metrics and patterns across Primate groups and species. Behav. Ecol. Sociobiol. (Print) **76** , 50. (10.1007/s00265-022-03133-5)

[35] Csárdi G , Nepusz T . 2006 The Igraph software package for complex network research. Inter. J. Complex Syst. **1695** , 1–9 (https://igraph.org).

[36] Sosa S , Sueur C , Puga‐Gonzalez I . 2021 Network measures in animal social network analysis: their strengths, limits, interpretations and uses. Methods Ecol. Evol. **12** , 10–21. (10.1111/2041-210X.13366)

[37] Sueur C , Petit O , De Marco A , Jacobs AT , Watanabe K , Thierry B . 2011 A comparative network analysis of social style in macaques. Anim. Behav. **82** , 845–852. (10.1016/j.anbehav.2011.07.020)

[38] Newman MEJ . 2006 Modularity and community structure in networks. Proc. Natl Acad. Sci. USA **103** , 8577–8582. (10.1073/pnas.0601602103) 16723398 PMC1482622

[39] Pasquaretta C *et al* . 2014 Social networks in primates: smart and tolerant species have more efficient networks. Sci. Rep. **4** , 7600. (10.1038/srep07600) 25534964 PMC4274513

[40] Neumann C , Fischer J . 2023 Extending bayesian elo-rating to quantify the steepness of dominance hierarchies. Methods Ecol. Evol. **14** , 669–682. (10.1111/2041-210X.14021)

[41] Friard O , Gamba M . 2016 BORIS: a free, versatile Open‐Source Event‐Logging software for Video/Audio coding and live observations. Methods Ecol. Evol. **7** , 1325–1330. (10.1111/2041-210X.12584)

[42] Scheider L , Liebal K , Oña L , Burrows A , Waller B . 2014 A comparison of facial expression properties in five Hylobatid species: facial expressions in Hylobatids. Am. J. Primatol. **76** , 618–628. (10.1002/ajp.22255) 24395677

[43] Wolak ME , Fairbairn DJ , Paulsen YR . 2012 Guidelines for estimating Repeatability. Methods Ecol. Evol. **3** , 129–137. (10.1111/j.2041-210X.2011.00125.x)

[44] RStudio Team . 2020 Rstudio: integrated development environment for R.

[45] Zeileis A , Hothorn T . 2002 Diagnostic checking in regression relationships. R. News. **2** , 7–10.

[46] Maechler M , Rousseeuw P , Struyf A , Hubert M , Hornik K . 2019 Hornik, cluster: Cluster analysis basics and extensions. See https://lirias.kuleuven.be/3999459?limo=0.

[47] Bender R , Lange S . 2001 Adjusting for multiple testing—when and how J. Clin. Epidemiol. **54** , 343–349. (10.1016/s0895-4356(00)00314-0) 11297884

[48] Thierry B , Singh M , Kaumanns W . 2004 Macaque societies: A model for the study of social organization. Cambridge: Cambridge University Press.

[49] Micheletta J , Engelhardt A , Matthews L , Agil M , Waller BM . 2013 Multicomponent and multimodal lipsmacking in crested macaques (Macaca nigra): Lipsmacking behavior in crested macaques. Am. J. Primatol. **75** , 763–773. (10.1002/ajp.22105) 23225489

[50] Clark PR , Waller BM , Agil M , Micheletta J . 2022 Crested Macaque facial movements are more intense and stereotyped in potentially risky social interactions. Phil. Trans. R. Soc. B **377** , 20210307. (10.1098/rstb.2021.0307) 35934960 PMC9358315

[51] Van Hooff J . 1972 A comparative approach to the Phylogeny of laughter and smiling. In Non-verbal commun. Cambridge, UK: Cambridge University Press.

[52] Tinbergen N . 1963 On aims and methods of Ethology. Z. F. T. **20** , 410–433. (10.1111/j.1439-0310.1963.tb01161.x)

[53] Amici F , Kulik L , Langos D , Widdig A . 2019 Growing into adulthood—a review on sex differences in the development of sociality across macaques. Behav. Ecol. Sociobiol. **73** , 18. (10.1007/s00265-018-2623-2)

[54] Whitehouse J . 2024 Facial expressivity in dominant macaques is linked to group cohesion. OSF https://osf.io/sx9fk/ 10.1098/rspb.2024.098439013427

[55] Whitehouse J , Clark PR , Robinson RL , Rees K , O’Callaghan O , Kimock C *et al* . 2024 Data from: Facial expressivity in dominant macaques is linked to group cohesion. Figshare. (10.6084/m9.figshare.c.7320025)39013427

